# Mechanistic Insight into Physical Activity Pleiotropy in Cancer Prevention

**DOI:** 10.1249/esm.0000000000000027

**Published:** 2024

**Authors:** Brooke M. Bullard, Brandon N. VanderVeen, Thomas D. Cardaci, James A. Carson, E. Angela Murphy

**Affiliations:** 1 Department of Pathology, Microbiology, & Immunology, School of Medicine, University of South Carolina, Columbia, SC, USA; 2 Department of Kinesiology and Sports Management, Sydney & JL Huffines Institute for Sports Medicine and Human Performance, Texas A&M University, College Station, TX, USA

**Keywords:** colonic environment, exercise, immune function, inflammation, metabolic dysregulation, sex hormones

## Abstract

Although improvements in prevention and screening have curbed the incidence of some cancers, the global burden of cancer is substantial and continues to grow. The sustained high prevalence of many cancers reveals the need for additional strategies to reduce occurrence. Observational studies have linked physical inactivity to the risk of 13 different cancers. Indeed, physical activity can reduce the occurrence of several cancers by more than 20%, whereas sedentary behavior can increase cancer risk. Thus, physical activity presents a viable lifestyle intervention to reduce the global burden of cancer, and current research efforts are focused on establishing the effective physical activity mode and intensity for cancer prevention. Preclinical cancer studies have provided insight into the mechanisms mediating these effects. There is growing evidence that physical activity can 1) reduce the risk of obesity and, by extension, metabolic dysregulation; 2) improve immune surveillance and reduce inflammation; 3) enrich the colonic environment by favoring beneficial microbes and reducing transit time; and 4) regulate sex hormones. This graphical review describes the current state of knowledge on the benefits of physical activity for cancer prevention and associated plausible mechanisms.

## INTRODUCTION

Global incidence is on the rise for many common cancers (1). Indeed, the World Health Organization’s International Agency for Research on Cancer predicts that we will see more than 35 million new cancer cases by 2050, a 77% increase from the estimated 20 million cases in 2022 (2). This precipitous increase reflects the growth and aging of the global population as well as changes in the occurrence and distribution of cancer-related risk factors, which incidentally can vary greatly by geographic region (1). Most cancers arise from a complex etiology involving genetic, environmental, and lifestyle factors, as well as their interactions (3). Given that a relatively low percentage of cancer cases can be attributed to genetic defects, many cancers can be described as preventable. Indeed, it is estimated that 30%–40% of cancers can be prevented through alterations in modifiable risk factors (4). Tobacco use, unhealthy diet, excess body weight, alcohol consumption, and physical inactivity account for these lifestyle risk factors, among others (5). Specifically for physical inactivity (i.e., sedentary behavior), there is an increased risk for developing breast, endometrial, ovarian, prostate, colon, and rectal cancers (5). On the other hand, being physically active is associated with decreased risk for bladder, breast, colon, endometrial, esophageal, adenocarcinoma, renal, and gastric cancers, with relative risk reductions ranging from approximately 10% to 20% depending on cancer type (4,6).

**Figure 1.**
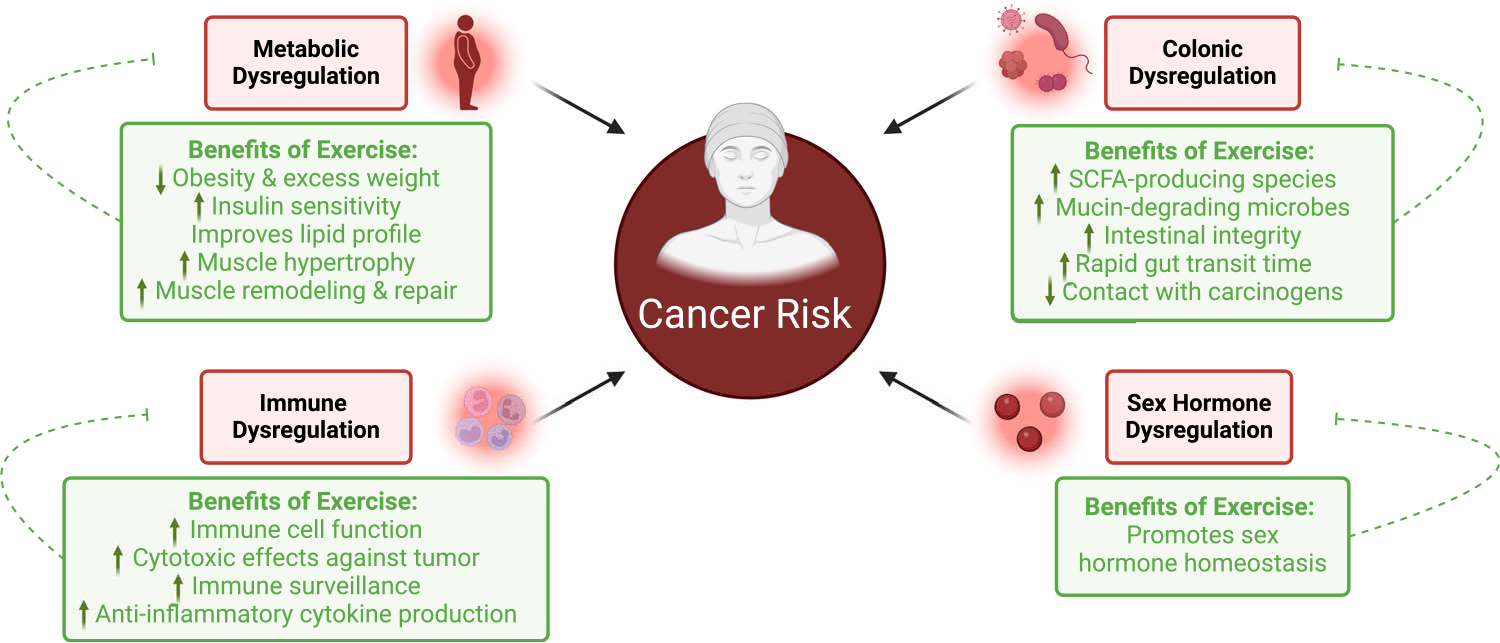
Mechanistic insight on the benefits of physical activity on cancer risk. SCFA, short-chain fatty acids.

The American Cancer Society recommends 150–300 min of moderate-intensity or 75–150 min of vigorous-intensity physical activity per week, or an equivalent combination, for cancer prevention (7). Despite these recommendations, epidemiological studies and clinical trials continue to focus on defining optimal physical activity prescriptions to reduce the risk of certain cancer types and for specific populations. Preclinically, studies are focused on providing insight into the pleiotropic mechanisms of physical activity on cancer risk. Indeed, the literature documents that physical activity can reduce obesity and associated metabolic dysregulation, improve immune and inflammatory profiles, enrich the colonic environment, and regulate sex hormones (Fig. 1), all of which have been associated with a decreased cancer risk. This graphical review will 1) describe the impact of these aforementioned factors on cancer risk, 2) discuss the mechanisms whereby physical activity can mediate improvements in these factors, and 3) identify gaps in the literature in the context of physical activity and cancer risk.

## OBESITY AND METABOLIC DYSREGULATION

Large cohort studies have linked obesity with increased risk for 13 cancer types, including endometrial, breast (postmenopausal), ovarian, colorectal, liver, and pancreatic, among others; controlled preclinical studies have substantiated these claims (8). In fact, obesity has been reported to account for ~20% of all cancer cases (9). Historically, body mass index (BMI), waist-to-hip circumference ratio, and weight gain have been utilized to categorize obesity. Although using BMI as a metric for obesity can be contentious (10), it has had utility for population-based investigations. Studies utilizing BMI support the conclusion that being overweight (BMI of 25–29.9 kg·m^−2^) or obese (BMI of >30 kg·m^−2^) is a cause of several cancers (8). Mechanistically, excess adipose tissue provokes “metabolic dysfunction,” a concept representing disordered metabolism on a continuum rather than a definite diagnosis. Although a consensus definition for metabolic dysregulation is currently lacking, the Metabolic Dysregulation and Cancer Risk Program at the National Cancer Institute was established to enhance understanding of metabolic dysregulation in the context of cancer risk with expectations for a forthcoming definition. Despite lacking a consensus definition, independent cancer risk factors attributed to defective metabolism in obese individuals include increased triglycerides, low high-density lipoprotein (HDL) cholesterol, high fasting blood glucose, and perturbations in the insulin/insulin-like growth factor (IGF) system. Epidemiology has linked serum triglyceride concentrations and low HDL cholesterol with risk for several cancers (11,12). Further, the IGF system, which is dysregulated in obesity, is a major determinant in the pathogenesis and progression of various cancers (13). Specifically, the IGF family consisting of insulin, IGF-1, and IGF-2 regulates cellular functions relevant to cancer, including mediating the growth and survival of cancer cells (i.e., Akt and mechanistic target of rapamycin (mTOR) signaling) (13). IGF’s actions are regulated by soluble IGF-binding proteins (IGFBP) and IGFBP proteases (13). High levels of insulin and IGF have been associated with risk for several obesity-related cancer types (14,15). Considering this, the utility of the IGF system to serve as a therapeutic target in cancer is being investigated and includes interventions involving IGF and IGF receptor antibodies and associated inhibitors.

**Figure 2.**
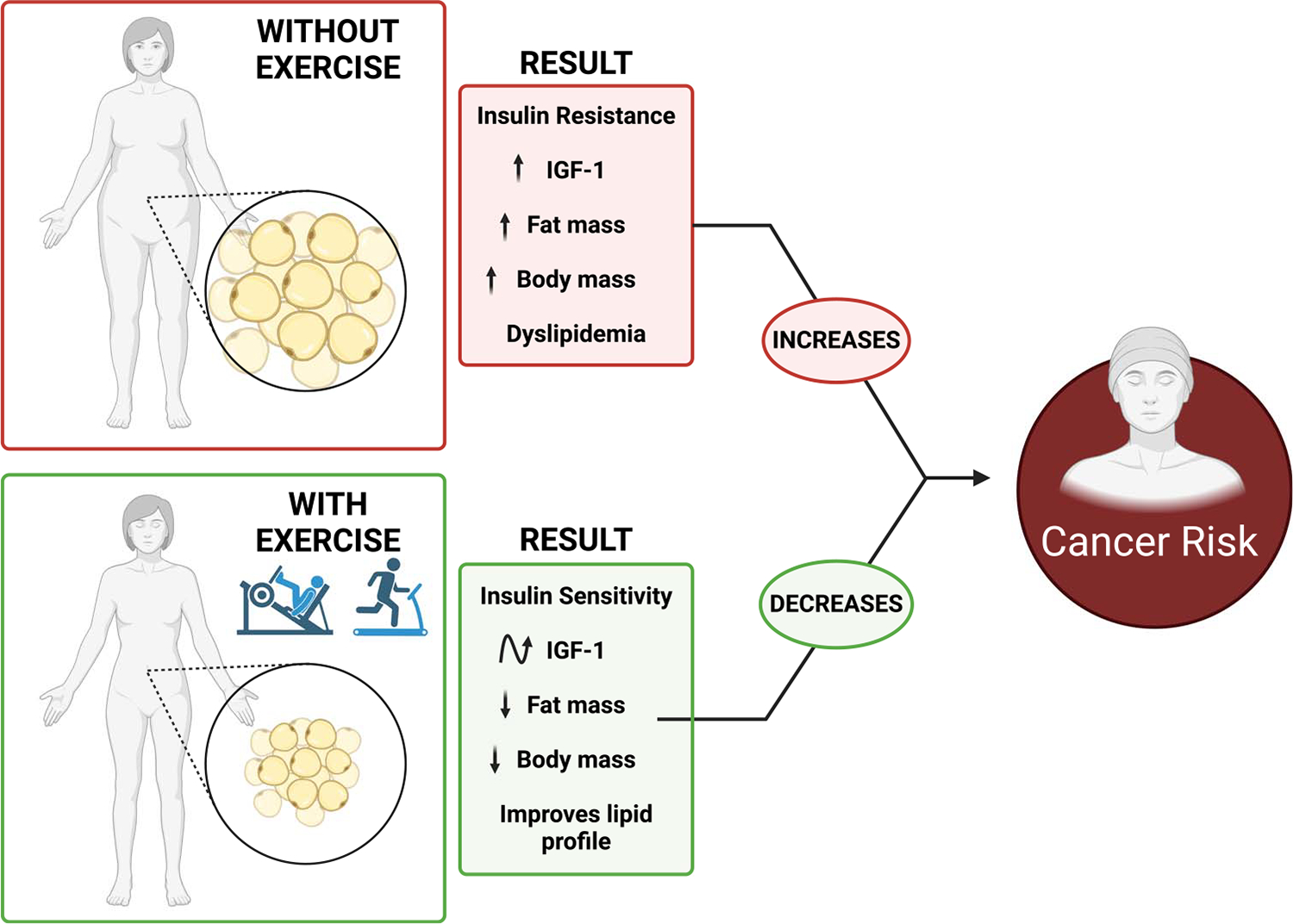
Impact of physical activity on obesity and metabolic dysregulation in the context of cancer risk. IGF, insulin/insulin-like growth factor.

A sedentary lifestyle in conjunction with a calorically dense diet is the main driver of obesity. Conversely, physical activity is widely acknowledged as a means to reduce obesity (i.e., fat mass) and consequently cancer risk (Fig. 2) (4). According to the American College of Sports Medicine, 150–250 min of moderate-to-vigorous activity weekly provides modest weight loss, with greater amounts (i.e., >250 min) required for clinically significant weight loss and consequential reversal of metabolic abnormalities. As such, in addition to reducing adiposity, physical activity can lower serum triglycerides and low-density lipoprotein (LDL) cholesterol levels and increase HDL cholesterol levels (16). The relationship between physical activity and insulin/IGF is more complex because they are dichotomous anabolic endocrine hormones with important physiological roles. For instance, although insulin is essential for the regulation of glucose homeostasis, hyperinsulinemia increases the risk of chronic diseases, including cancer. It is well established that physical activity can reduce plasma insulin levels, resulting in increased insulin sensitivity. Indeed, this response has been confirmed in cancer survivors following 150 min of weekly moderate-intensity aerobic activity (17). Many studies indicate a dose–response with higher energy expenditures and higher intensities producing greater benefits to insulin sensitivity, although these findings are not strictly unanimous (18). Further, both aerobic and resistance activity can improve insulin sensitivity, and it has been suggested that incorporating both forms of activity may be more efficacious than either mode alone (18). Interestingly, it appears that the benefits of physical activity on insulin sensitivity can occur independent of weight loss (18).

Although beneficial for growth/repair and regulation of muscle hypertrophy (19), high levels of IGF-1 are associated with increased cancer risk (4). Interestingly, the impact of physical activity on IGF-1 appears to be contingent on the mode of physical activity. Resistance activity has been shown to elevate IGF-1 concentrations, which tracks the physiological demand for growth/repair (20). Endurance activity, on the other hand, has been shown to decrease circulating IGF-1 levels (21). Indeed, IGF-1 levels appear to be primarily correlated with an energy deficit and resulting changes in body composition (22). However, it is important to note that there are inconsistencies in the literature concerning the IGF-1 response to physical activity; some studies report no changes in IGF-1 levels, suggesting that many factors can influence this response (e.g., age, sex, mode of physical activity, time of assessment, training level). Not much is known about the IGF-2 response, precluding any firm conclusions on this neglected insulin.

Physical activity is a well-established lifestyle modality to counter obesity and associated metabolic dysregulation, ultimately contributing to reduced cancer risk (4). Although the benefits of physical activity on weight loss and associated metabolic dysregulation are undisputed, the field continues to unravel the impact of mode, intensity, and duration of physical activity on various biomarkers associated with metabolic dysregulation. Further, it is interesting to note that the late Dr. Steven Blair has suggested a “fit but fat” paradox; although reports of changes in some biomarkers can occur with physical activity independent of weight loss (18), more work in this area is needed to understand changes dependent on and independent of weight loss. Finally, although guidelines from governing bodies exist for weight loss, personalized and sustainable approaches to physical activity are likely to have the greatest long-term benefits for managing weight and, by extension, metabolic dysfunction.

## IMMUNE SURVEILLANCE AND INFLAMMATION

The immune system plays an important role in cancer risk reduction by recognizing and eliminating abnormal tumor cells. It is well recognized that natural killer (NK) cells, phagocytic macrophages, neutrophils, dendritic cells, and lymphocytes all have tumor suppressor roles (23); one of the most potent therapeutic strategies against cancer has been tightly linked with the immunological profile of the tumor microenvironment. For instance, the higher the number of infiltrating cytotoxic T cells and NK cells in the tumor, the better the prognosis (23). Thus, strategies to enhance various immune responses against cancer are currently being exploited in the clinic to include immunotherapy approaches, which rely on the presence of “hot” tumors (i.e., high T-cell infiltration) for successful outcomes. Unfortunately, tumor cells have developed strategies to incapacitate immune cell cytotoxicity by promoting the expression of inhibitory receptor ligands as well as an immune suppressive environment (e.g., promotion of tumor-associated macrophages).

Likewise, inflammation is associated with the development and malignant progression of most cancer types and accordingly represents an attractive strategy for cancer prevention. Inflammation is a necessary response to tissue injury that involves a multifactorial network of chemical signals, which are initiated and augmented following the recruitment and infiltration of white blood cells (e.g., monocytes). Typically, a self-limiting inflammatory response is terminated following tissue repair; however, if the inflammatory response is unregulated and consequently sustained, it can promote malignant growth and tumor initiation (24). In fact, epidemiology indicates that at least 20% of all cancer cases are due to a state of chronic inflammation driven by obesity, infection, autoimmune diseases, alcohol consumption, and other factors (24). A wide range of inflammatory mediators (e.g., cytokines, chemokines, growth factors, prostaglandins, free radicals) can promote tumor development (24). Sources of these mediators include innate (e.g., macrophages, neutrophils, NK cells, dendritic cells) and adaptive (e.g., T cells, B cells) immune cells, as well as fibroblasts, myocytes, adipocytes, endothelial cells, and even cancer cells themselves (24). Targeting inflammatory processes continues to represent an attractive strategy for cancer control.

**Figure 3.**
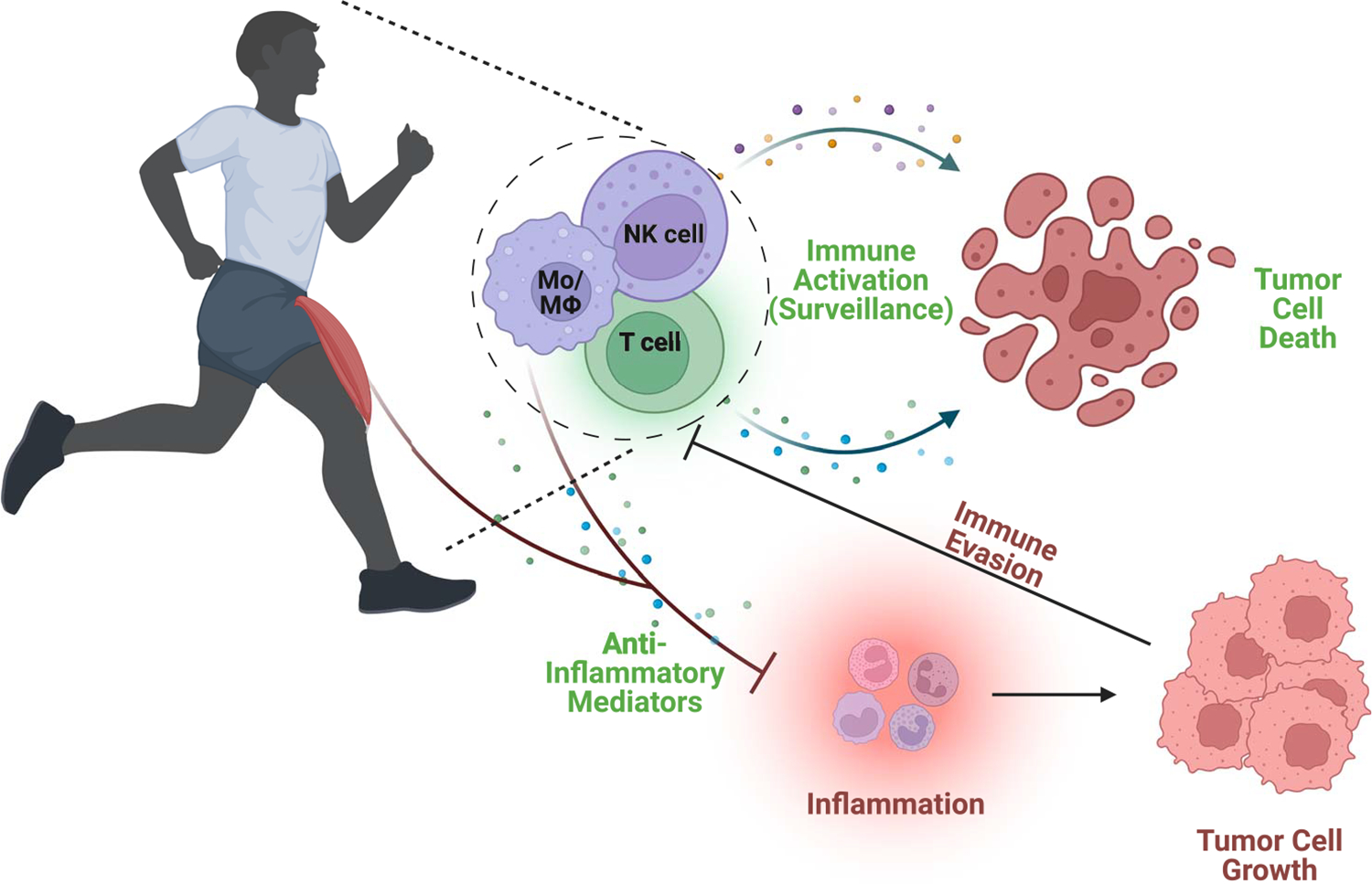
Physical activity improves immune surveillance and reduces inflammation resulting in decreased risk of cancer. Mф, macrophages; Mo, monocytes; NK, natural killer.

Physical activity can confer favorable effects on the immune system and the inflammatory response (Fig. 3). It is generally well accepted that physical activity improves the number and functioning of several immune cells with tumor suppressor roles, including NK cells, macrophages, neutrophils, dendritic cells, and lymphocytes (23). The deployment of activated immune cells following a physical activity stimulus can increase immune surveillance, thereby reducing cancer risk. For instance, NK cells, which are rapidly mobilized into circulation during physical activity, can exert a direct cytotoxic effect on tumor cells and promote an elevated T-cell response via the production of cytokines and chemokines (23). Similarly, physical activity can increase the cytotoxicity of macrophages, which is consistent with a reduction in their tumor-promoting properties (23). Likewise, physical activity can confer beneficial effects on T-cell functioning in the context of cancer (23). However, it is essential to note that these relationships are complex and multifaceted. Indeed, although it is well accepted that acute and chronic aerobic-based physical activity results in significant immunomodulation regarding the distribution, function, and activity of immune cells, the magnitude of these effects is influenced by the duration and intensity of the physical activity. Although investigators continue to explore the optimum physical activity prescription to maximize immune surveillance in cancer prevention, we can agree that the immune system is highly responsive to physical activity, resulting in diverse and extensive immunomodulation that can be harnessed for cancer control.

In addition to improving immune surveillance, physical activity is consistently reported to reduce inflammation (25). Many factors can contribute to this response, including reduced fat mass, increased production and release of anti-inflammatory cytokines from contracting skeletal muscle, and reduced expression of toll-like receptors (TLR) on macrophages and monocytes (25). Adipose tissue is a rich reservoir for chronic low-grade inflammation, given the accumulation of macrophages and subsequent formation of crown-like structures. Thus, it is no surprise that the reduction of adipose tissue mass associated with physical activity tracks with a reduction in proinflammatory cytokines, in direct contrast to obesity (25). Physical activity can result in a marked increase in interleukin-6 (IL-6) from skeletal muscle, the magnitude of which is highly dependent on the mode, intensity, and duration of activity as well as the individual’s training status. The transient increase in IL-6 promotes an anti-inflammatory environment mediated by the release of IL-10 and IL-1 receptor antagonists (25). Furthermore, physical activity has been reported to reduce the expression of TLR on immune cells, rendering them less responsive to an inflammatory stimulus (25). Together, there is convincing evidence that regular physical activity can promote an anti-inflammatory state that may contribute to its documented antitumor benefits.

Although the benefits of physical activity on immune surveillance and inflammation are undisputed, contention remains regarding the optimal mode, duration, and intensity to confer such benefits. In general, moderate-intensity activity leads to immune response enhancement, whereas high-intensity activity or overtraining can suppress the immune system via stress hormone responses. A better understanding of the cellular and molecular outcomes associated with physical activity mode, intensity, and duration will allow for improved physical activity recommendations for cancer prevention.

## COLONIC ENVIRONMENT

Over the last decade, we have witnessed an explosion of literature on the importance of gut health in cancer prevention. These studies have focused on gut microbes, which are malleable to lifestyle modifications and have an essential role in regulating health. Inappropriate host–microbiota interactions have been documented to trigger a wide range of inflammatory chronic diseases, including cancer (26). Indeed, gut microbes have been reported to play a role in cancer initiation and prevention (26). In fact, approximately 20% of cancers may be due to both imbalances in the gut microbiome and the involvement of pathogens (26). Disturbances in gut microbes can increase cancer predisposition through multiple mechanisms, including the production of oncogenic toxins and metabolites, promotion of inflammation, and modulation of immune responses (26). This research area has focused, to date, on gastrointestinal cancers, including colorectal cancer and pancreatic cancer, with more limited evidence for cancers remote (e.g., breast cancer) from the gastrointestinal tract. Although there is strong evidence to indicate that select bacteria (e.g., *Fusobacterium nucleatum* in colorectal cancer and *Porphyromonas gingivalis* in pancreatic cancer) are involved in tumorigenesis (26), it should be stressed that a direct causal role in cancer has not yet been established. It is also important to note that longitudinal studies have not examined the link between gut microbes and cancer risk in human subjects. Colon transit time has also been linked to cancer and specifically colorectal cancer; the association between constipation or infrequent bowel movements and the risk of colorectal cancer has been documented (26). The mechanisms for this phenomenon have been linked to an increased exposure to carcinogens in the intestinal mucosa (27).

**Figure 4.**
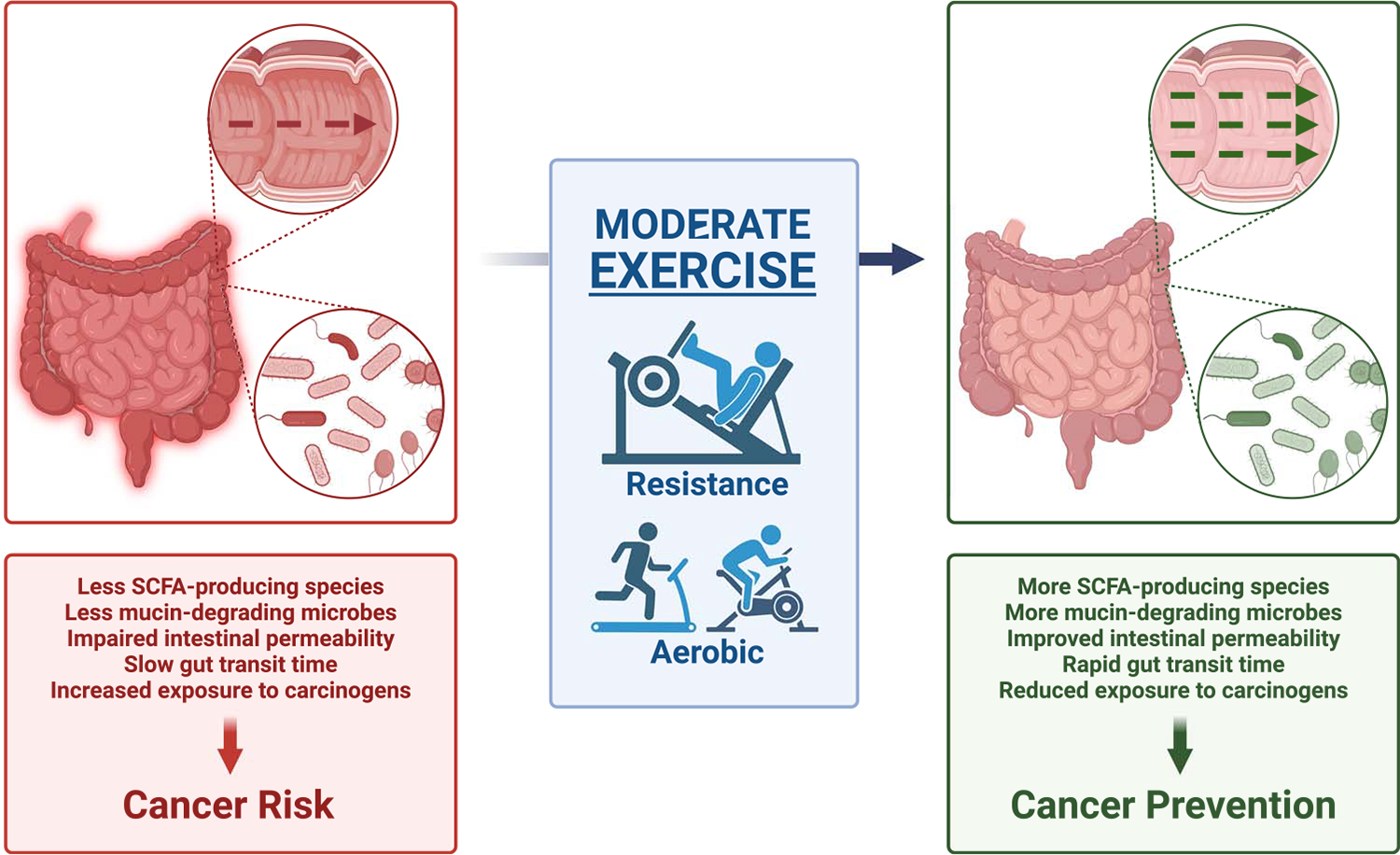
Benefits of physical activity on gut health as related to cancer risk. SCFA, short-chain fatty acids.

Physical activity has been reported to promote gut health (Fig. 4). A recent review described physical activity as a powerful modulator of the composition and functional alterations in the gut microbiota, which are reversed with training cessation (28). Proposed mechanisms for these effects include increased production of short-chain fatty acids, improved intestinal integrity, enhanced mucin-degrading microbes, and increased capacity for energy harvest (28). Mechanistic studies in mice using fecal microbiome transfer show that these benefits can be conferred (29). A preclinical study in mice reported that physical-activity–mediated improvements in metabolic and inflammatory profiles are transmissible via fecal microbiota transfer (29), highlighting the therapeutic potential of gut microbes. Interestingly, the mode, intensity, and duration of physical activity appear to influence alterations to gut microbes (30). For instance, in preclinical studies, voluntary wheel running, and forced treadmill running have been reported to differentially alter the microbiome (31). More specifically, the different modalities had distinctive effects on community diversity, structure, and taxonomy (31). In clinical studies, when compared to aerobic activity, the gut microbiota appeared to be less malleable to the stimulus of resistance activity (30). However, as noted in a relatively recent systematic review, not all changes to the gut microbiota with aerobic activity were viewed as favorable. Indeed, several studies reported adverse effects, including gastrointestinal distress, decreased microbial diversity, and increased intestinal permeability (30). Interestingly, it appeared that the favorable effects of physical activity on gut microbes were more evident in nontrained individuals and the detrimental effects were documented in athletes, which is most likely related to the level (intensity and duration) of training (i.e., overtraining) (30). Therefore, moderate levels of physical activity may be optimal for gut health, modeling the J-shaped curve formulated by Dr. David Nieman to describe the relationship between physical activity intensity and the risk of acquiring an upper respiratory tract infection (32).

Further adding to the complexity of this relationship, the literature indicates that the effects of physical activity on microbiota may be segment-dependent and more extensive in the distal gut (33). It has been noted that physical activity can alter the microbiome at more than one intestinal site, with differences observed within the feces and cecum located in the distal colons of mice (31). Although the physical-activity–microbiota interaction is currently understudied in the context of cancer, some of the aforementioned changes to gut microbes with physical activity are inverse to changes observed with cancer. Thus, gut microbes may be contributing to the beneficial effects of physical activity on cancer risk. Physical activity can also result in a more rapid transit time—the time during which carcinogens within the gastrointestinal tract are in contact with the intestinal mucosa—thereby suppressing carcinogenesis (34). Interestingly, more time spent on light-intensity activity was optimal for more rapid colonic and whole-gut transit time (34), providing additional support for the concept that moderate physical activity may be more beneficial to gut health than more strenuous physical activity.

Alterations to gut microbe signatures and decreased transit time may contribute to the benefits of physical activity on cancer risk. The current literature supports differential effects on gut microbes and intestinal transit time depending on physical activity mode, intensity, and duration. However, a more definitive understanding of how variations in physical activity can influence these parameters remains to be determined, especially in the context of cancer risk. Although some physical-activity-induced alterations to gut health may, at least in part, theoretically explain the inverse association reported between physical activity and cancer risk, this relationship remains speculative until such time that causation can be inferred.

## SEX HORMONE REGULATION

Despite their essential physiological roles, sex hormones have been associated with an increased risk for certain cancers, particularly reproductive cancers, and a possible reduced risk for some nonreproductive cancers. Sex hormones, mainly including estrogens, progesterone, and androgens, are potent signaling molecules produced by the endocrine system that regulate physiological processes. In the context of cancer, sex hormones have proliferative properties that can promote the growth of cancer cells. However, contention surrounds the relationship between sex hormones and cancer risk, which is likely driven by a lack of understanding of the intricacies involved. Indeed, menopausal status, age of menarche, contraceptive use, family history, type of cancer (e.g., hormone sensitivity), hormone therapy (including when it was started and the specific type/combination), BMI, diet, and other factors can impact this relationship. For instance, epidemiological studies have documented increased rates of breast cancer in the premenopausal years (35), whereas estrogen is associated with a lower incidence of invasive breast cancer in postmenopausal women (36). Further, testosterone has traditionally been associated with prostate cancer risk, but this notion is currently being challenged (37). When it comes to nonreproductive cancers, there is literature to suggest a benefit of estrogen on cancer risk; for example, estrogen is associated with a reduced risk of colorectal cancer (38). Additional work in this area is needed to truly understand the complexity surrounding sex hormones and cancer risk, as well as the mechanisms involved.

**Figure 5.**
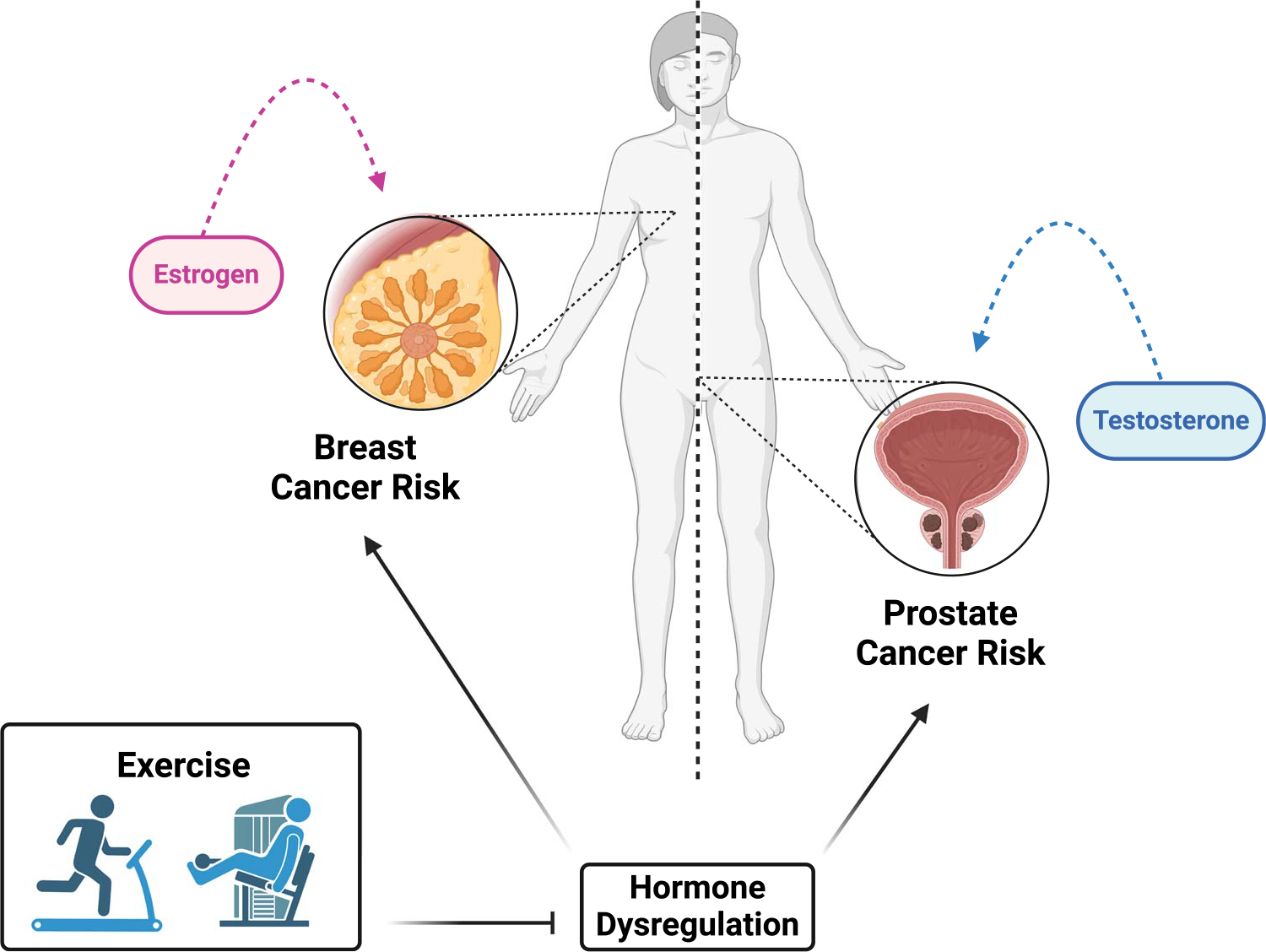
Physical activity may regulate sex hormones to reduce risk of hormone-sensitive cancers.

Despite the contention surrounding sex hormones and cancer risk, studies have postulated that the benefits of physical activity on cancer risk are mediated, at least in part, via the regulation of sex hormones (Fig. 5). This theory is based on the hypothesis that higher levels of sex hormones increase the risk for hormone-sensitive cancers (i.e., breast and prostate). In females, physical activity has been linked to lower levels of circulating estrogen, presumably due to disruption of menstrual function or delayed onset of menarche (39). A recent systematic review and meta-analysis of the effects of physical activity on sex hormones found that physical activity leads to a decrease in estradiol, free estradiol, progesterone, testosterone, free testosterone, and dehydroepiandrosterone (39). Although randomized controlled trials (RCT) utilizing aerobic and resistance-type physical activities, as well as their combination, were included in the assessment, no clear evidence emerged as to what type of physical activity has the greatest influence on sex hormones (39). However, it should be noted that most RCT involve aerobic physical activity (39). What is less clear are the mechanisms driving this decrease. As discussed above, regular physical activity reduces weight gain, which may reduce circulating estrogen levels because adipose tissue is the main source of estrogen in postmenopausal women (40). Indeed, the available literature suggests that reduced fat mass mediates, at least in part, the reduction in sex hormones with physical activity (39). One caveat is that estrogen has been reported to reduce inflammation (41); thus, decreasing estrogen levels may lead to imbalances or perturbations in other physiological systems, including those that can drive cancer (i.e., inflammation). An improved understanding of the mechanistic pathways that may underlie the physical activity–breast cancer relationship is warranted to strengthen causal inference.

A recent review on the impact of physical activity on testosterone levels revealed that a temporary surge in testosterone occurs following both endurance and resistance physical activity, but does not appear to be sustainable beyond several minutes, nor does it seem to impact basal levels (42); therefore, by extension, it would not impact cancer risk. Conversely, in the context of exhaustive endurance activity, decreases in testosterone have been documented, which may contribute to the recognized physical-activity-mediated reduction in prostate cancer risk (42). However, there is high variability across studies due to differences in physical activity protocols, study populations (e.g., age, obesity status), and timing of assessment (42). Further work in this area is needed to fully understand the role of testosterone, if any, in the relationship between physical activity and prostate cancer risk.

Although the benefits of physical activity on cancer risk are undisputed, the contribution of sex hormones to this relationship has not been established. Indeed, a dearth of mechanistic evidence precludes any firm conclusions. Additionally, and not surprisingly, most of the literature is focused on implications for hormone-sensitive cancers, whereas studies on other cancers are lacking. Further work in this area is needed to truly understand the implications of sex hormones on the relationship between physical activity and cancer risk.

## CONCLUSION

Cancer incidence is increasing in the United States in parallel with escalating levels of unhealthy, yet modifiable, behaviors (e.g., physical inactivity, poor diet). Current evidence indicates that physical activity can reduce overall cancer risk, with decreases of 10%–20% reported in the literature. Although a lifelong approach to physical activity is likely to confer the greatest benefits to health, including those that extend beyond cancer risk, implementing an active lifestyle at any stage is likely to reduce cancer risk, given that even a single bout of physical activity can influence many of the proposed mechanisms (e.g., gut microbes, inflammation, immune function). Physical activity intensity appears to be a critical factor in relative risk reduction, but gaps remain in understanding the mechanistic basis for the relationship. Furthermore, we know less about the impact of physical activity mode because most research has focused on endurance activity, with far fewer studies examining the relationship between resistance activity and cancer risk, implying that additional work in this area is needed. Controlling weight status, improving the immune and inflammatory profile, enhancing the colonic environment, and regulating sex hormone levels are leading candidate mechanisms whereby physical activity mediates beneficial effects on cancer risk and should be further explored using mechanistic approaches, especially in the context of cancer type where evidence is limited for certain cancers.
